# RELATIONSHIP BETWEEN PHYSICAL ACTIVITY AND DEPRESSIVE SYMPTOMS IN STROKE SURVIVORS: A CROSS-SECTIONAL STUDY OF 1,140 INDIVIDUALS

**DOI:** 10.2340/jrm.v57.41272

**Published:** 2025-01-08

**Authors:** Yihao WANG, Jiali CHEN, Yanwei ZOU, Mengshu YANG, Xiaoyun KONG, Ling WANG, Jingyuan XUE, Ci DONG

**Affiliations:** 1 Department of Neurology, The First Hospital of Hebei Medical University, Hebei, China; 2 Department of Medicine, Shinshu University, Nagano-ken, Japan

**Keywords:** stroke, depression, physical activity, NHANES data, propensity score matching

## Abstract

**Objectives:**

To investigate the relationship between physical activity and depressive symptoms in stroke survivors.

**Design:**

A cross-sectional study utilizing National Health and Nutrition Examination Survey (NHANES) 2007–2018 data, employing propensity score matching to control for confounders.

**Patients:**

1,140 stroke survivors from NHANES, assessing depressive symptoms through the Patient Health Questionnaire-9 (PHQ-9) conducted via family interview or a mobile examination centre examination.

**Methods:**

PA was surveyed concurrently with the PHQ-9, categorized into vigorous, moderate, and moderate-to-vigorous intensities. Propensity score matching was used to match participants based on their activity levels, and the relationship between physical activity and depressive symptoms was analysed by logistic regression.

**Results:**

Among all the subjects, 225 individuals had significant depressive symptoms. If vigorous-intensity PA duration is longer than 75 min (odds ratio [OR] = 0.41, 95% CI 0.21–0.75) or longer than 150 min (OR = 0.42, 95% CI 0.19–0.85), and moderate-intensity physical activity duration is longer than 150 min (OR = 0.59, 95% CI 0.38–0.90) or between 150 and 300 min (OR = 0.36, 95% CI 0.15–0.77), and moderate-to-vigorous PA duration is greater than 150 min (OR = 0.61, 95% CI 0.40–0.91) or exceeding 300 min (OR = 0.50, 95% CI 0.31–0.78), this might be associated with lower depressive symptoms.

**Conclusion:**

Regular physical activity, particularly of moderate or higher intensity, is associated with milder depressive symptoms in stroke survivors, suggesting the potential for non-pharmacological intervention.

## LAY ABSTRACT

This study explores how physical activity impacts depression in stroke survivors. It finds that moderate to vigorous exercise is linked to fewer depressive symptoms, suggesting that an active lifestyle may aid mental health recovery post-stroke. Tailored exercise plans could be key to managing depression for stroke survivors.

Stroke remains the second leading cause of death and the third leading cause of disability-adjusted life-years lost in the world (1). According to the World Stroke Organization 2022 report (2), the burden of stroke increased significantly between 1990 and 2019. Depression is one of the most common complications after stroke. According to 2022 Best Available Evidence, the incidence of depression after stroke at any time is 29% and the cumulative probability up to 52% within 5 years (3). Depressive symptoms are prevalent among stroke survivors, with significant implications for their overall well-being (4). Not only does depression diminish the self-care capabilities of stroke survivors, consequently lowering their quality of life seriously, but it also contributes to a decrease in their long-term physical activity levels (5). This hinders recovery and increases the risk of the patient dying (6, 7). Furthermore, the presence of depression is linked to an increased likelihood of experiencing a secondary stroke, thereby imposing a substantial burden on patients, their families, and society at large (8–11). Addressing depression’s impact on survivors is crucial for improving long-term outcomes and reducing societal implications.

Moderate physical activity has been shown to have a protective effect against cerebrovascular accidents, such as strokes (12). Additionally, moderate physical activity may prevent depression onset (8, 13). The World Health Organization (WHO) also pointed out that 75 to 150 min of vigorous activity or 150 to 300 min of moderate-intensity activity per week is beneficial to preventing cerebrovascular diseases and reducing the occurrence of depressive symptoms (14). Nevertheless, most studies on exercise intervention for post-stroke depression use a single form of exercise, although their conclusions all imply that physical activity has a positive effect on relieving depression in stroke patients (15, 16), but related studies have also found that physical activity has no clear impact on long-term depressive symptoms in stroke survivors (17, 18). The study by Liang et al. confirmed that not all types of intensity exercise can reduce the occurrence of depressive symptoms (19), and research by Xing et al. shows that, compared with high-intensity activities, moderate and low-intensity exercise only can play a neuroprotective role (20). Moreover, stroke survivors frequently exhibit varying degrees of physical activity impairment due to age and disease (21), which not only affects their ability to perform physical activities but may also increase the patient’s risk of depression (22). In the aforementioned studies, very few studies fully assessed the physical function of stroke survivors and incorporated it into their analysis, which limits the applicability of their conclusions with limited significance for stroke survivors with widespread physical mobility impairment. Furthermore, most existing studies are underpowered. Thus, the relationship between exercise and depression after stroke is difficult to clarify.

This study aims to improve the quality of research by using a larger sample size, more scientific sampling methods, and controlling for observable confounding variables to better explain the effects of activity of different intensity and duration on depressive symptoms in stroke survivors. The findings of this study will inform the development of evidence-based physical activity recommendations for stroke survivors.

## METHODS

### Study population

This cross-sectional study used 6 cycles of data from the National Health and Nutrition Examination Survey (NHANES), conducted by the National Centre for Health Statistics of the Centers for Disease Control and Prevention in the United States, from 2007 to 2018. The NHANES data, which are publicly available on the official website (23), include interviews and physical examinations and are designed to evaluate the nutrition and health status of American children and adults. The study comprised a family interview and a mobile examination centre (MECs) examination. Only participants who self-reported a previous stroke diagnosis were included, while those who answered “No”, “Do not know”, or refused to answer the question were excluded.

**Fig. 1:**
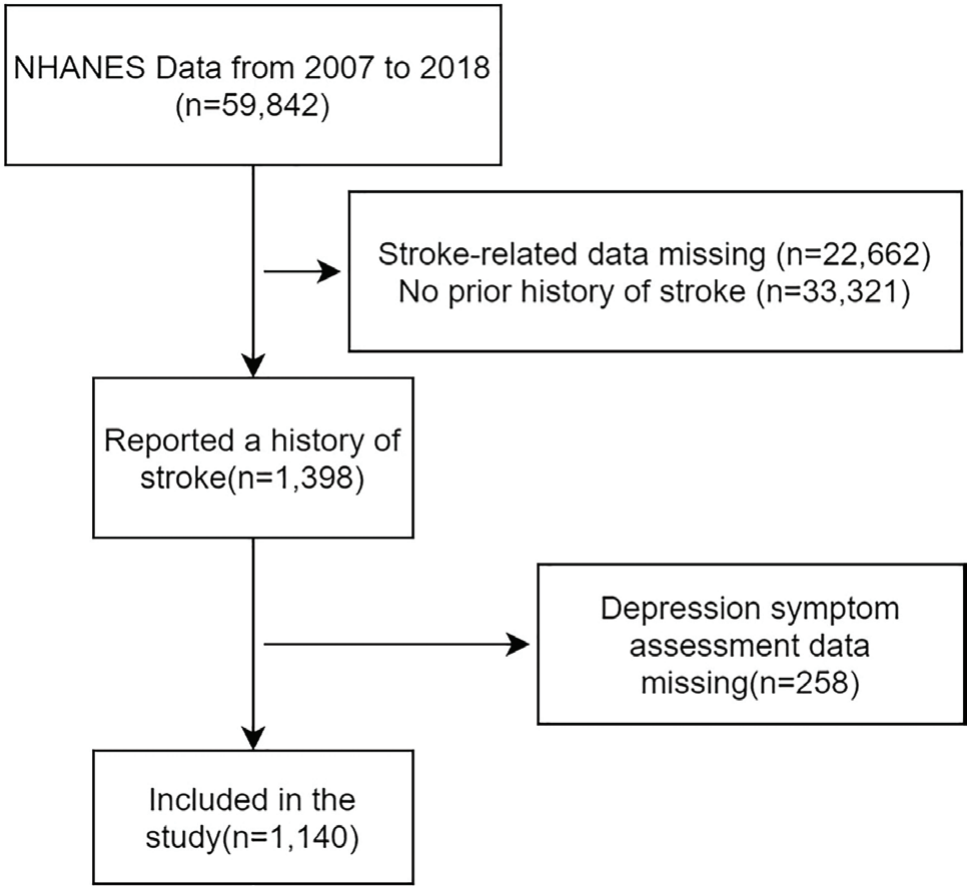
Process for conducting participant screening.

Following these criteria, there are 59,842 individuals in these cycles. Initially, 1,398 individuals answered “Yes”, and we excluded participants who did not provide depression symptom measurements (*n* = 258). Finally, data from 1,140 participants were included in the analysis, all of whom were over the age of 18. The study’s flowchart is shown in Fig. 1. The implementation of the NHANES project was approved by the ethics review committee of the National Center for Health Statistics, and written informed consent was obtained from each participant. The NHANES protocol details are available on the Centers for Disease Control (CDC) website (24).

### Assessment of physical activity

Physical activity (PA) is typically composed of physical measurements from accelerometers, direct observations, and subjects’ self-reports. The data collection in NHANES is nationwide, hence most participants are missing data from accelerometers and direct observations. Therefore, we chose short-term self-reported PA as the primary outcome for PA. The Physical Activity Survey of NHANES 2007–2018 measured the time spent on work, leisure activities, and sedentary activities. The survey includes variables such as moderate recreational activity or work over the past 30 days, vigorous recreational activity or work over past 30 days, walking or cycling over past 30 days, and hours watching TV or videos or using a computer in the past 30 days. Additionally, the survey calculates the frequency and daily average duration of each activity within 7 days (23). The duration of weekly activity was calculated, and the minutes of moderate-intensity PA (MPA), vigorous-intensity PA (VPA), and moderate-to-vigorous PA (MVPA) were determined (MVPA-min = 2×VPA-min+MPA-min) (19). According to the recommendations of the WHO in 2020 (14), we categorize weekly VPA duration into 3 groups: < 75 min, 75~150 min, and > 150 min. Similarly, MPA time per week is divided into 3 groups: < 150 min, 150~300 min, and > 300 min. MVPA time per week is also divided into 3 groups: < 150 min, 150~300 min, and > 300 min. At the same time, we calculated the participants’ MVPA intensity by multiplying the VPA duration by 2, divided by the MVPA duration. The overall MVPA intensity was then categorized into 4 dimensions: 0% to 25%, 26% to 50%, 51% to 75%, and 76% to 100% (19). Dummy variables were set for each dimension for analysis.

### Ascertainment of depression symptoms

The NHANES data from 2007 to 2018 utilized the Patient Health Questionnaire-9 (PHQ-9) to evaluate individuals’ symptoms of depression. The PHQ-9 has sound factor structure, reliability, and validity, and has been validated for use in primary care for depressive symptoms (25). The questionnaire consists of 9 questions, each with response options ranging from “not at all” to “almost every day”, scored from 0–3. A total score of 0–27 is possible, with a score of > 9 indicating the presence of depressive symptoms (26).

### Assessment of covariates

The standardized questionnaire collected age, gender, race/ethnicity (Mexican American, other Hispanic, non-Hispanic White, non-Hispanic Black, and other Race – including Multi-Racial), educational attainment (less than 9th Grade, 9th–11th Grade, High School Grad/GED or equivalent, Some College or AA degree, College Graduate or above), poverty-to-income ratio (PIR), and marriage status (married, widowed, divorced, separated, never married, and living with partner). Additionally, the survey included questions about self-reported hypertension, diabetes, and hyperlipidaemia (ever told you had high blood pressure/diabetes/high cholesterol level), as well as smoking history (smoked more than 100 cigarettes in life; whether you currently smoke) and alcohol consumption (including the average number of drinks per day in the past 12 months, with excessive drinking defined as more than 4 drinks). The survey also collected information on the age at which the stroke was first reported, height, and weight, which were used to calculate BMI.

Physical function was assessed based on these 5 areas: (*i*) Lower extremity mobility (including 3 factors: walking for a quarter of a mile, difficulty; walking up 10 steps, difficulty; stooping, crouching, kneeling, difficulty), (*ii*) General physical activity (6 factors: lifting or carrying, difficulty; standing up from armless chair, difficulty; standing for long periods, difficulty; sitting for long periods, difficulty; reaching up over head, difficulty; grasping/holding small objects, difficulty), (*iii*) Activities of daily living (ADLs; 4 factors: walking between rooms on same floor; getting in and out of bed, difficulty; using fork, knife, drinking from cup; dressing yourself, difficulty), (*iv*) Instrumental ADL (IADL; 3 factors: managing money, difficulty; household chores, difficulty; preparing meals, difficulty), (*v*) Leisure and social activities (3 factors: going out to movies/events, difficulty; attending social events, difficulty; leisure activity at home, difficulty).

Each item was scored from 0 to 3 based on the response options: no difficulty, some difficulty, much difficulty, or unable to do the activity (27). We calculated the overall physical function score for each participant by adding the scores across the 19 items (27). The total score ranges from 0 to 57 points, with higher scores indicating worse physical activity ability.

### Statistical analyses

The data were analysed using R software version 4.3.2 (R Foundation for Statistical Computing, Vienna, Austria). Since the NHANES database uses complex multi-stage probability sampling (28), the weighted data sample can represent the characteristics of the surveyed population. Weighted means and standard errors are thus used to describe continuous variables, and weighted frequencies are used to describe categorical variables. Taking into account some issues such as the general demographic information of some participants obtained from MECs testing, the weight is selected as “WTMEC2YR” and divided by 3 to obtain the weight of the 6-cycle sample (29). To reduce the differences in baseline data of participants between different exercise behaviours and intensities, we used the propensity score matching method (PSM) to reduce the impact of confounding factors and propensity score regression (PS score instead of covariates for regression analysis) to test the sensitivity of the match. We conducted a logistic regression analysis to assess the impact of exercise behaviour on depression among stroke survivors, with balanced exercise status as the independent variable, the propensity score (PS score) as the covariate, and the presence or absence of depressive symptoms as the dependent variable (30). Regression results were displayed as odds ratio (OR) and 95% CI and *p* < 0.05 (two-sided) was considered statistically significant in all tests.

## RESULTS

### Characteristics of population

The screening process for subjects is depicted in Fig. 1. Table I displays the weighted and unweighted statistics of the participants’ general demographic information and related covariates. Among the participants, 225 exhibited obvious signs of depression, while 915 did not. The results indicate that, apart from the observed statistical differences in taking insulin, physical function, smoke, and stroke age, there were no significant differences between the depressed and non-depressed groups in other variables. The weighted data showed a significant difference from the unweighted data, and the weighted data are more representative.

**Table I:** Participant-weighted and unweighted general profile

Level	Weighted			Unweighted		
	Depression		*p*-value	Depression		*p*-value
	No	Yes		No	Yes	
*n*	4,750,546	1,133,718.1		915	225	
Gender (%)						
Male	2,197,011.5 (46.2)	428,622.8 (37.8)	0.244	471 (51.5)	92 (40.9)	0.006*
Female	2,553,534.5 (53.8)	705,095.3 (62.2)		444 (48.5)	133 (59.1)	
Age, mean (SD)	65.57 (13.44)	58.19 (13.16)	0.084	66.70 (12.60)	60.05 (12.97)	< 0.001*
Race/Ethnicity (%)						
Mexican American	205,286.3 (4.3)	59,325.3 (5.2)	0.536	75 (8.2)	21 (9.3)	0.627
Other Hispanic	145,862.6 (3.1)	43,181.4 (3.8)		56 (6.1)	18 (8.0)	
Non-Hispanic White	3,303,741.8 (69.5)	772,897.5 (68.2)		450 (49.2)	114 (50.7)	
Non-Hispanic Black	705,133.8 (14.8)	186,802.8 (16.5)		264 (28.9)	55 (24.4)	
Other Race - Including Multi-Racial	390,521.5 (8.2)	71,511.1 (6.3)		70 (7.7)	17 (7.6)	
Education level (%)						
Less than 9th Grade	419,161.3 (8.8)	119,901.9 (10.6)	0.150	121 (13.2)	34 (15.1)	0.001*
9–11th Grade	738,415.6 (15.5)	208,837.0 (18.4)		165 (18.0)	54 (24.0)	
High School Grad/GED or equivalent	1,467,358.0 (30.9)	400,353.7 (35.3)		263 (28.7)	62 (27.6)	
Some College or AA degree	1,173,593.2 (24.7)	340,552.6 (30.0)		233 (25.5)	65 (28.9)	
College Graduate or above	952,017.8 (20.0)	64,072.9 (5.7)		133 (14.5)	10 (4.4)	
Marital status (%)						
Married	2,542,780.4 (53.5)	467,197.4 (41.2)	0.113	455 (49.7)	81 (36.0)	0.001*
Widowed	936,762.9 (19.7)	191,719.6 (16.9)		181 (19.8)	45 (20.0)	
Divorced	570,958.7 (12.0)	211,542.6 (18.7)		137 (15.0)	41 (18.2)	
Separated	101,058.8 (2.1)	64,351.5 (5.7)		32 (3.5)	14 (6.2)	
Never married	333,154.1 (7.0)	135,707.3 (12.0)		72 (7.9)	33 (14.7)	
Living with partner	265,831.2 (5.6)	63,199.6 (5.6)		38 (4.2)	11 (4.9)	
Family PIR, mean (SD)	2.42 (1.48)	1.81 (1.22)	0.357	2.13 (1.38)	1.61 (1.10)	< 0.001*
Been told had high blood pressure (%)						
Yes	3,573,508.8 (75.2)	822,066.7 (72.5)	0.159	706 (77.2)	170 (75.6)	0.673
No	1,177,037.2 (24.8)	311,651.4 (27.5)		209 (22.8)	55 (24.4)	
Been told had high cholesterol level (%)						
Yes	2,911,890.3 (61.3)	745,226.3 (65.7)	0.124	563 (61.5)	148 (65.8)	0.271
No	1,838,655.6 (38.7)	388,491.8 (34.3)		352 (38.5)	77 (34.2)	
Been told had diabetes (%)						
Yes	1,455,643.5 (30.6)	398,744.5 (35.2)	0.362	308 (33.7)	84 (37.3)	0.218
No	3,106,870.9 (65.4)	720,367.1 (63.5)		566 (61.9)	136 (60.4)	
Borderline	188,031.5 (4.0)	14,606.5 (1.3)		41 (4.5)	5 (2.2)	
Had diabetes age, mean (SD)	55.99 (9.63)	49.79 (10.68)	0.104	55.95 (10.19)	51.27 (10.48)	< 0.001*
Taking insulin (%)						
Yes	467,792.8 (9.8)	247,535.5 (21.8)	0.002*	111 (12.1)	42 (18.7)	0.014*
No	4,282,753.2 (90.2)	886,182.7 (78.2)		804 (87.9)	183 (81.3)	
Take diabetic pills (%)						
Yes	3,557,937.1 (74.9)	735,635.5 (64.9)	0.098	681 (74.4)	154 (68.4)	0.083
No	1,192,608.9 (25.1)	398,082.6 (35.1)		234 (25.6)	71 (31.6)	
Drink (%)						
≤ 2 cups per day	4,154,990.7 (87.5)	901,562.4 (79.5)	0.118	809 (88.4)	180 (80.0)	0.005*
> 2 but <4 cups per day	280,744.7 (5.9)	88,245.3 (7.8)		51 (5.6)	18 (8.0)	
≥ 4 but <14 cups per day	305,956.1 (6.4)	143,910.4 (12.7)		54 (5.9)	27 (12.0)	
≥ 14 cups per day	8,854.5 (0.2)	0.0 (0.0)		1 (0.1)	0 (0.0)	
Physical function, mean (SD)	6.38 (4.52)	8.52 (4.58)	0.015*	6.51 (4.47)	8.43 (4.34)	< 0.001*
Smoke (%)						
Not at all	2,050,700.0 (43.2)	330,381.9 (29.1)	0.008*	366 (40.0)	74 (32.9)	< 0.001*
Smoked over 100 cigarettes but not now	17,05,247.0 (35.9)	305,560.2 (27.0)		347 (37.9)	61 (27.1)	
Still smoking	994,598.9 (20.9)	497,776.0 (43.9)		202 (22.1)	90 (40.0)	
Stroke age, mean (SD)	56.33 (16.85)	48.42 (15.20)	0.038*	57.64 (15.66)	50.58 (14.94)	< 0.001*
Weight, mean (SD)	81.55 (20.74)	85.65 (23.20)	0.435	81.27 (19.41)	84.49 (23.07)	0.032*
Height, mean (SD)	167.68 (10.46)	167.97 (10.32)	0.725	167.99 (10.24)	168.04 (10.40)	0.954
BMI, mean (SD)	28.90 (6.52)	30.29 (7.24)	0.367	28.73 (6.09)	29.97 (7.34)	0.009*
MVPA (%)						
< 150 min	2,858,504.0 (60.2)	799,172.8 (70.5)	0.422			
≥ 150 min and ≤ 300 min	493,149.8 (10.4)	93,795.2 (8.3)				
< 300 min	1,398,892.2 (29.4)	240,750.1 (21.2)				
VPA (%)						
< 75min	3,941,753.5 (83.0)	1,022,783.0 (90.2)	0.054			
≥ 75min and ≤ 150 min	113,184.9 (2.4)	46,429.9 (4.1)				
< 150 min	695,607.7 (14.6)	64,505.2 (5.7)				
MPA (%)						
< 150 min	3,117,222.2 (65.6)	864,664.3 (76.3)	0.348			
≥ 150 min and ≤ 300 min	580,439.8 (12.2)	51,386.8 (4.5)				
< 300 min	1,052,884.0 (22.2)	217,667.0 (19.2)				
VPA/MVPA (%)						
0%~25%	1,513,084.3 (64.3)	320,325.2 (70.4)	0.610			
26%~50%	162,266.3 (6.9)	39,997.7 (8.8)				
51%~75%	31,3232.6 (13.3)	40,072.9 (8.8)				
76%~100%	363,317.6 (15.4)	54,296.3 (11.9)				
Walked or bicycled (%)						
Yes	710,402.9 (15.0)	178,812.2 (15.8)	0.644			
No	4,040,143.1 (85.0)	954,905.9 (84.2)				
Vigorous work (%)						
Yes	686,507.0 (14.5)	140,065.0 (12.4)	0.499			
No	4,059,057.3 (85.4)	993,653.1 (87.6)				
Vigorous recreational activity (%)						
Yes	331,497.5 (7.0)	12,575.6 (1.1)	0.002*			
No	4,419,048.5 (93.0)	1,121,142.5 (98.9)				
Moderate work (%)						
Yes	1,397,122.8 (29.4)	328,200.4 (28.9)	0.860			
No	3,345,308.4 (70.4)	805,517.7 (71.1)				
Moderate recreational activity (%)						
Yes	1,402,246.5 (29.5)	246,233.0 (21.7)	0.452			
No	3,340,247.7 (70.3)	887,485.1 (78.3)				
Watch TV or use computer, mean (SD)	4.83 (2.14)	5.11 (2.78)	0.699			

* *p*-value less than 0.05.

### Physical activity situation

Table I displays the physical activity levels. In cases without PSM match, the only difference between the depressed and non-depressed groups is whether they engage in vigorous recreational activity (*p* = 0.002). There are no differences in specific activities or the intensity of VPA, MPA, MVPA, or time spent watching TV or using a computer.

### Propensity score matching result

PSM matching was performed to determine participation in each specific activity and to calculate the duration of each activity. Table II presents the results of the regression analysis conducted with exercise levels as the independent variable and depressive symptoms as the dependent variable in the datasets after matching in each group. Figs S1–S8 show the balance of each covariate before and after matching. The analysis indicates that the balance of each variable before and after matching is relatively good, enhancing the regression results’ reliability. The analysis results show that if VPA duration is longer than 75 min (OR = 0.41, 95% CI 0.21–0.75) or the VPA duration is longer than 150 min (OR = 0.42, 95% CI 0.19–0.85), and the MPA duration is longer than 150 min (OR = 0.59, 95 % CI, 0.38–0.90) or the MPA duration is between 150 and 300 min (OR = 0.36, 95% CI 0.15–0.77), and the MVPA duration is greater than 150 min (OR = 0.61, 95% CI 0.40–0.91) or greater than 300 min (OR = 0.50, 95% CI 0.31–0.78), this is possibly associated with non-significant depressive symptoms, and sensitivity of the PS regression test is good. The regression results suggest that VPA lasting 75 and 150 min promotes depressive symptoms in stroke survivors compared with other durations. However, the PS regression results show no statistical significance and poor sensitivity. Additionally, the performance of certain types of activities was assessed and MVPA intensity had no statistical significance.

**Table II:** Propensity score matching and propensity score regression results

Variable	Matched individuals (*n*)	Method	PSM				PS			
			OR	95% CI		Pr(>|z|)	OR	95% CI		Pr(>|z|)
				2.50%	97.50%			2.50%	97.50%	
Walked or bicycled										
Yes or no	418	2:1 nearest neighbour matching with replacement	0.778	0.442	1.353	0.378	0.807	0.493	1.306	0.388
Vigorous work										
Yes or no	374	2:1 nearest neighbour matching with replacement	0.659	0.371	1.151	0.148	0.716	0.424	1.190	0.204
Vigorous recreational activity										
Yes or no	142	2:1 nearest neighbour matching with replacement	0.202	0.031	0.910	0.055	0.272	0.060	0.896	0.051
Moderate work										
Yes or no	679	2:1 nearest neighbour matching with replacement	0.905	0.592	1.379	0.644	0.959	0.652	1.405	0.829
Moderate recreational activity										
Yes or no	627	2:1 nearest neighbour matching with replacement	0.621	0.375	1.014	0.060	0.654	0.411	1.027	0.068
Watch TV or use computer										
>4 h per day or not	405	2:1 nearest neighbour matching with replacement	0.956	0.540	1.672	0.876	0.978	0.581	1.625	0.932
VPA										
<75 min or ≥75 min	385	2:1 nearest neighbour matching with replacement	0.407	0.214	0.747	0.005*	0.492	0.277	0.850	0.013*
<75 min and >150 min or 75~150 min	1,140	Optimal full matching	2.940	1.055	7.634	0.031*	2.044	0.861	4.528	0.087
<150 min or ≥150 min	326	2:1 nearest neighbour matching with replacement	0.416	0.194	0.852	0.020*	0.442	0.228	0.817	0.012*
MPA										
<150 min or ≥150 min	724	2:1 nearest neighbour matching with replacement	0.585	0.375	0.902	0.016*	0.637	0.422	0.951	0.029*
<150 min and >300 min or 150–300 min	278	2:1 nearest neighbour matching with replacement	0.355	0.150	0.771	0.012*	0.426	0.195	0.869	0.024*
<300 min or ≥300 min	555	2:1 nearest neighbour matching with replacement	0.714	0.431	1.167	0.184	0.755	0.472	1.194	0.235
MVPA										
<150 min or ≥150 min	789	2:1 nearest neighbour matching with replacement	0.607	0.404	0.906	0.015*	0.622	0.428	0.899	0.012*
<150 min and >300 min or 150–300 min	276	2:1 nearest neighbour matching with replacement	1.509	0.682	3.328	0.306	1.635	0.823	3.244	0.158
<300 min or ≥300 min	652	2:1 nearest neighbour matching with replacement	0.495	0.310	0.778	0.003*	0.539	0.355	0.809	0.003*
VPA/MVPA										
>25% or 0%~25%	726	2:1 nearest neighbour matching with replacement	0.914	0.604	1.375	0.667	0.899	0.609	1.321	0.589
<26% and >50% or 26%~50%	97	2:1 nearest neighbour matching with replacement	0.284	0.042	1.389	0.148	0.508	0.151	1.463	0.233
<51% and >75% or 51%~75%	175	2:1 nearest neighbour matching with replacement	0.601	0.204	1.630	0.331	0.580	0.241	1.291	0.199
<76% or 76%~100%	212	2:1 nearest neighbour matching with replacement	0.685	0.284	1.573	0.383	0.714	0.333	1.465	0.369

* *p*-value less than 0.05.

## DISCUSSION

Based on an analysis of the NHANES database from 2007 to 2018, our study focused on investigating the correlation between physical activity and depressive symptoms among stroke survivors in the United States. Our sample consisted of 1,140 participants, and potential confounding variables were reduced by propensity score matching. While we did not find a significant association between moderate or vigorous work/exercise and depressive symptoms, a notable association emerged between total physical activity and depressive symptoms. Specifically, engaging in more than 75 min of vigorous exercise or more than 150 min of moderate-intensity exercise per week was associated with less severe depressive symptoms in stroke survivors. In addition, participation in 150 to 300 min of moderate-intensity activity per week also showed a potential association with milder depressive symptoms.

Analysis of the demographic information on the 1,140 participants revealed no significant difference in the mean weighted age of the participants in the 2 groups. However, participants in the depressed group had a lower mean age than those in the nondepressed group, suggesting that there may be a potential effect of age on depressive symptoms in stroke survivors. The proportion of females in the depressed group was also higher than in the non-depressed group, which is consistent with previous studies (31) but not statistically significant (32). Unsurprisingly, the depressed group had poorer physical functioning. However, it remains challenging to determine whether the decrease in somatic functioning directly contributes to increased depression or if decreased physical activity is a contributing factor to depressive symptoms. Also, the depressed group showed higher rates of insulin use and higher rates of smoking, suggesting that insulin use and smoking may be associated with more severe depressive symptoms. In addition, like the mean age results, the age at first stroke in the depressed group is younger. This could have been linked somehow to the dramatic changes in their social roles and functions. Therefore, it is essential to control for potential confounding variables, such as insulin use, physical functioning scores, smoking status, and age at first stroke, in order to accurately assess the impact of physical activity on participants’ depressive symptoms.

Although there have been many studies on exercise treatment programmes for depressive symptoms in stroke survivors (33), there remains a paucity of reliable evidence on the optimal duration and intensity of exercise for this population. Based on NHANES data, this study utilized a complex multi-layer sampling method to ensure a relatively representative population of stroke survivors. By employing propensity score matching, we were able to control for common confounders that are known to be associated with depression in stroke survivors. This approach allowed us to assess the relationship more accurately between physical activity and stroke survivors’ depression, providing valuable insights for potential interventions and support strategies for this population. It is noteworthy that the relationship between activity intensity and duration and the risk of depressive symptoms in stroke survivors was not uniform. This finding differs from that of Pearce et al., who studied the general population (8). This confirms that stroke survivors wanting to reduce the risk of depressive symptoms through exercise require a different intensity and duration of exercise than the general population. Based on the analysis results, it is evident that the intensity of exercise, whether moderate or vigorous, is not of relative significance regarding the depressive symptoms experienced by stroke survivors. Instead, it is the duration of exercise that appears to be an important factor strongly associated with depressive symptoms. The results for the duration of VPA and MVPA duration are similar, meaning that doing more than 75 min of VPA per week or more than 150 min of MVPA per week is associated with a low risk of depressive symptoms. This is consistent with a 1-year randomized controlled trial by Krawcyk et al. The results of this study are similar, meaning that VPA which exceeds the duration of exercise recommended by the WHO can still promote the improvement of mental well-being in stroke survivors (34). However, this conclusion should nonetheless take into account that stroke survivors are generally older and have varying degrees of somatic activity dysfunction, so rational exercise strategies should still be tailored to the individual survivor, but at least this finding demonstrates the minimum number of hours of exercise that can reduce the risk of depressive symptoms in stroke survivors. We also calculated the optimal classification value using the ROC curve to determine a cut-off value, which was found to be close to the WHO recommendation, as shown in Figs S9–S11.

It is interesting to note that the protective effect of exercise on depressive symptoms does not disappear when the exercise duration exceeds the WHO recommendations of VPA 150 min and MVPA 300 min. The research by Ashizawa et al. suggests that minutes of low-intensity PA (LPA) performed early after stroke may also help reduce the incidence of depression after discharge in stroke survivors, but the duration of MVPA shown in the results has no significant impact on depressive symptoms (17), which is different from the results obtained in this study. This difference may be due to the relatively low average weighted age of the participants included in this study (the weighted average age of the participants in this study was 64.15±13.69, while the average age of the participants in Ashizawa and colleagues’ study was 71.2±7.8), and the average physical function score was relatively low (6.79±4.60), which was caused by less severe limitations in physical function. However, the analysis results of the MPA duration in this study suggest that when the MPA duration is between 150 and 300 min, there is a correlation between the low depressive symptoms of stroke survivors and activity, but when the MPA duration exceeds 300 min, this correlation disappears. There are certain differences in the conclusions that any degree of physical activity can have a beneficial effect on depressive symptoms in stroke survivors (11, 15–17, 34). The findings indicate that, for individuals who have experienced a stroke, the assumption that increased exercise, particularly moderate-intensity exercise, is always beneficial may require further examination. Nevertheless, no significant statistical difference was observed in the intensity analysis of MVPA, which differs from the findings of Liang et al. (19). This may be because the reduced physical function of stroke survivors limits their long-term performance. Further research is needed to better understand these underlying mechanisms.

The study acknowledges limitations in the precision of its results. It is crucial to acknowledge at the outset that, as a cross-sectional study, it is not possible to derive a definitive causal relationship. Consequently, the results presented here must be interpreted with caution. One limitation of the study is the reliance on self-reported data for stroke history and physical activity. This may introduce the potential for recall bias. Nevertheless, given the profound impact of stroke on an individual’s life, the potential for recall bias may be relatively limited (12). However, this limitation must take into account the recall bias that may result from cognitive impairments in stroke patients. In addition, a significant amount of information was excluded from the participant screening process due to missing primary study information, which may have had some impact on the representativeness of the results. Another limitation is lack of details on stroke type and severity, the sequence of occurrence between stroke events, and the emergence of depressive symptoms. Additionally, the related content of medication and treatment and potential biases in participant selection and analysis methods may have affected the outcomes. Further research is needed to explore the relationship between exercise and depression among stroke survivors in more diverse samples.

In conclusion, the analysis of the NHANES database from 2007 to 2018 reveals a significant correlation between total physical activity and depressive symptoms among stroke survivors. However, the study also underscores the importance of considering potential confounding variables such as insulin use, physical functioning scores, smoking status, and age at first stroke to accurately assess the impact of physical activity on depressive symptoms in this population. While the study’s cross-sectional design limits the establishment of a definitive causal relationship, it does provide valuable insights into the potential of physical activity as a non-pharmacological approach to managing depressive symptoms in stroke survivors.

## Supplementary Material

RELATIONSHIP BETWEEN PHYSICAL ACTIVITY AND DEPRESSIVE SYMPTOMS IN STROKE SURVIVORS: A CROSS-SECTIONAL STUDY OF 1,140 INDIVIDUALS
