# Supplementary material for: RELATIONSHIP BETWEEN PHYSICAL ACTIVITY AND DEPRESSIVE SYMPTOMS IN STROKE SURVIVORS: A CROSS-SECTIONAL STUDY OF 1,140 INDIVIDUALS
Source: J Rehabil Med. 2025 Jan 8;57:41272. doi: 10.2340/jrm.v57.41272 (PMC11744704; doi:10.2340/jrm.v57.41272)
Supplement: RELATIONSHIP BETWEEN PHYSICAL ACTIVITY AND DEPRESSIVE SYMPTOMS IN STROKE SURVIVORS: A CROSS-SECTIONAL STUDY OF 1,140 INDIVIDUALS [file JRM-57-41272-s1.pdf]

Supplementary material has been published as submitted. It has not been copyedited, or typeset by Journal of Rehabilitation Medicine

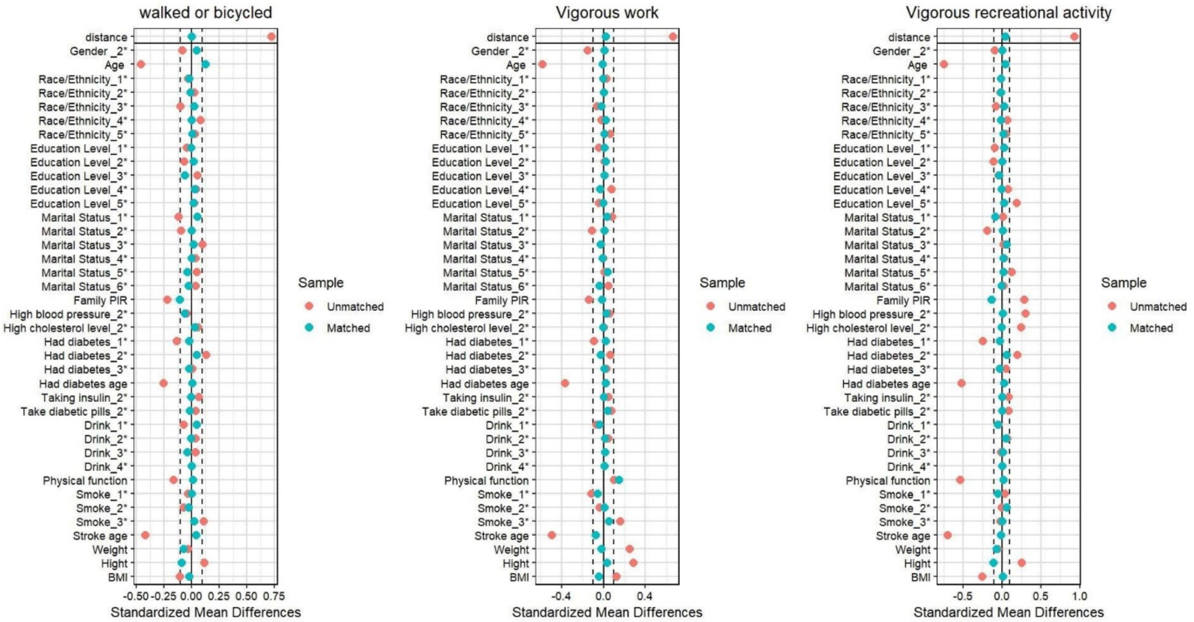

Fig. S1: The balance before and after matching1

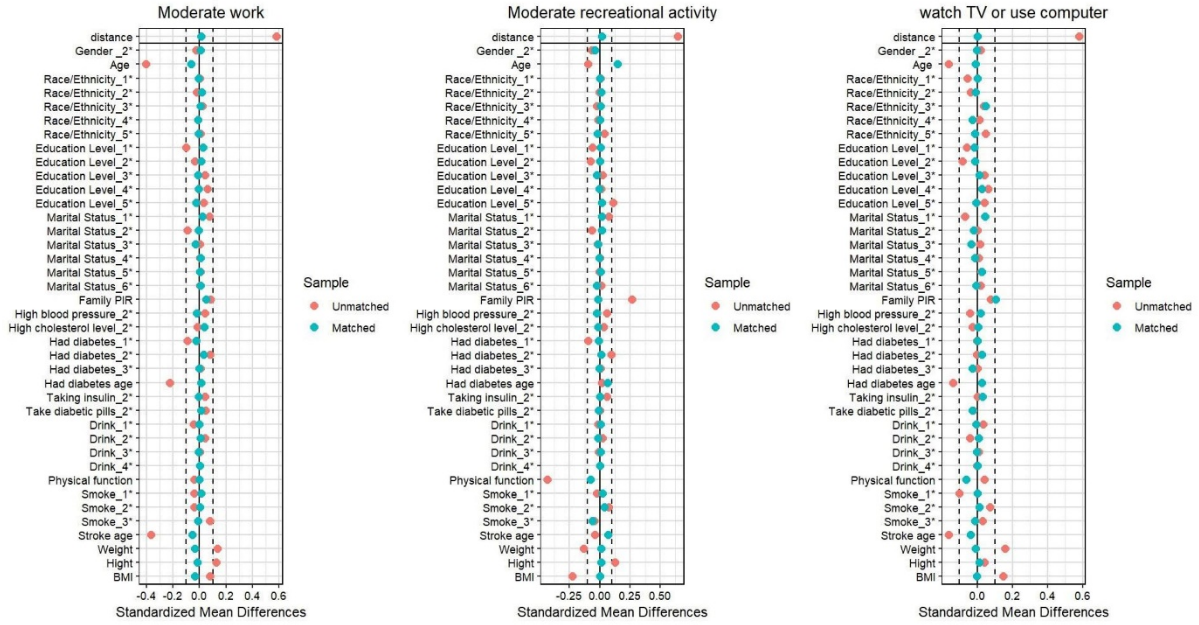

Fig. S2: The balance before and after matching2

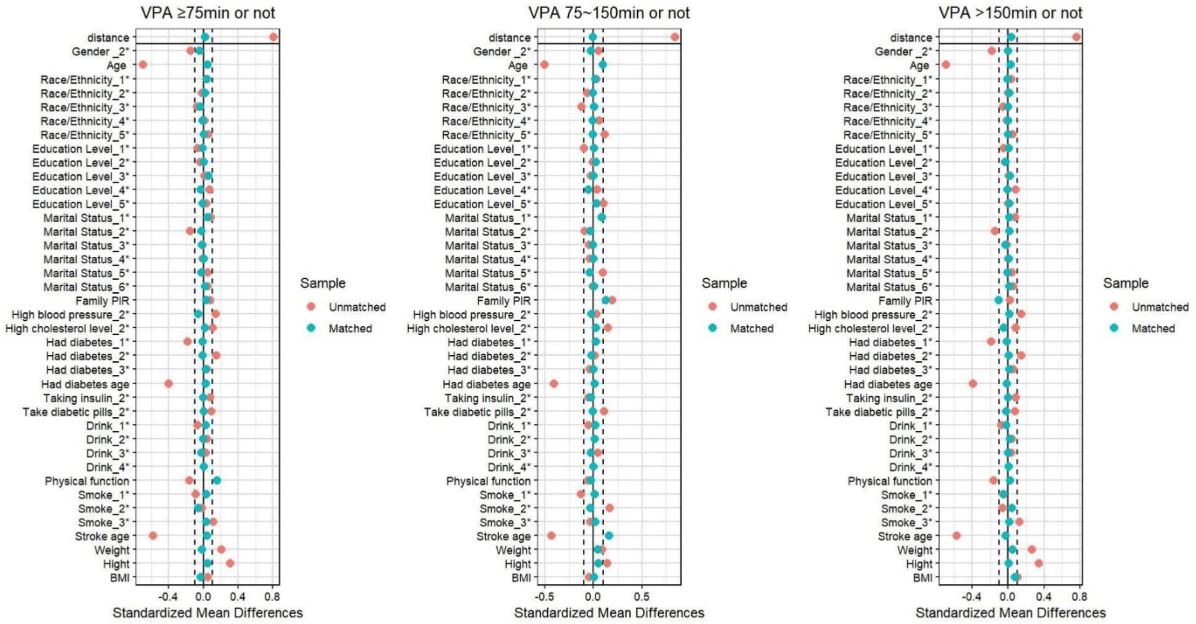

Fig. S3: The balance before and after matching3

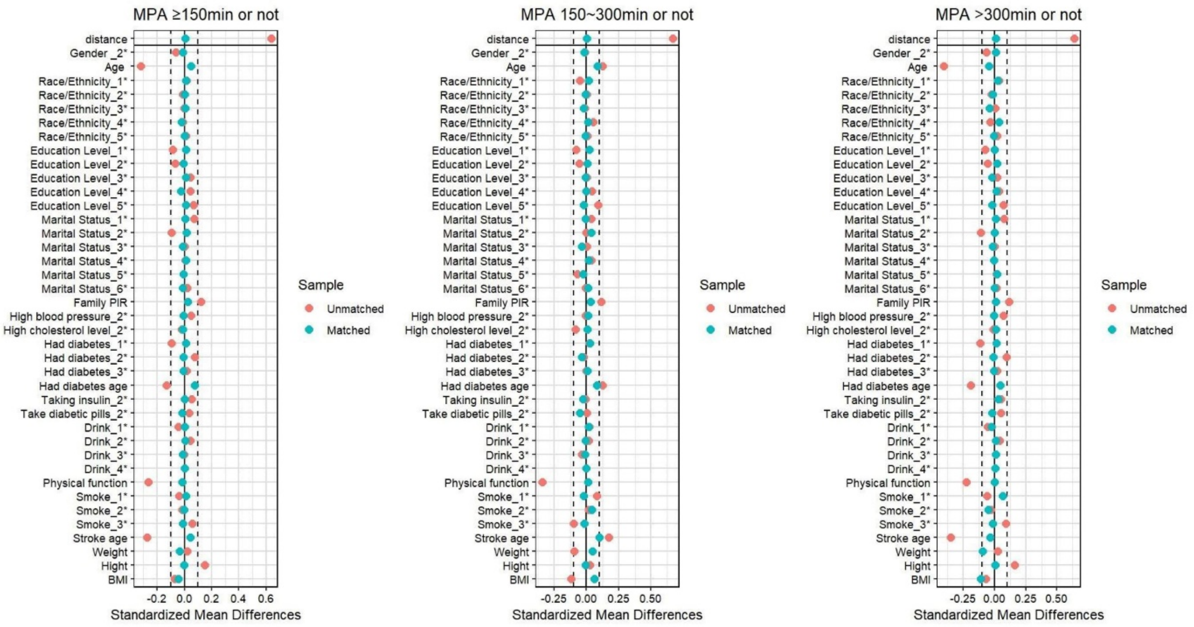

Fig. S4: The balance before and after matching4

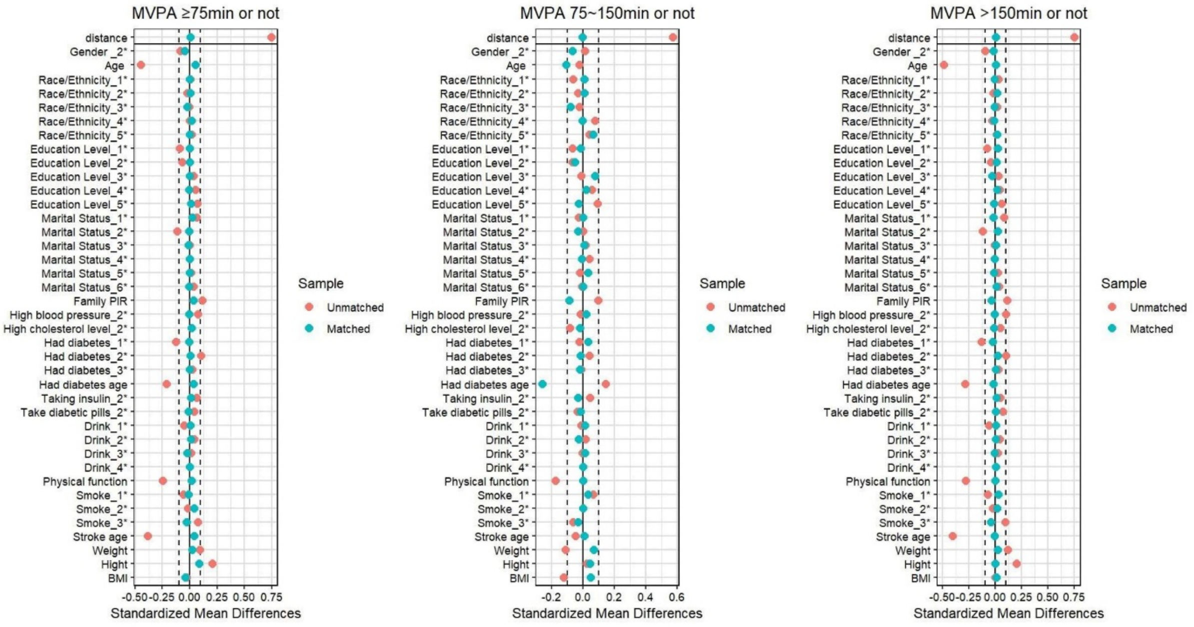

Fig. S5: The balance before and after matching5

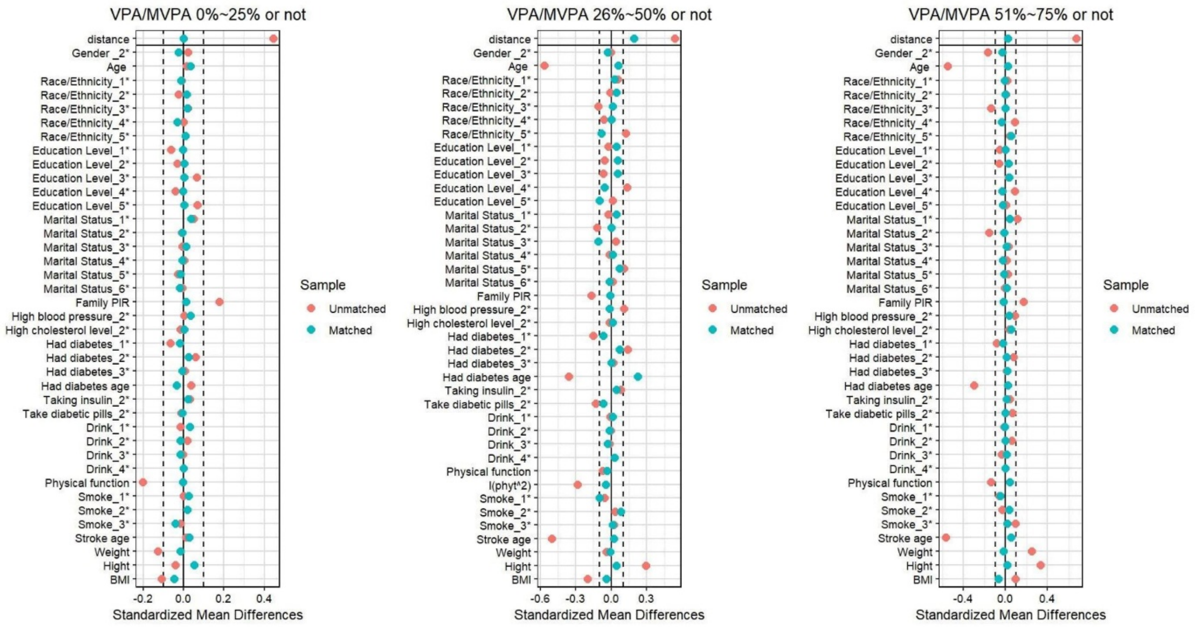

Fig. S6: The balance before and after matching6

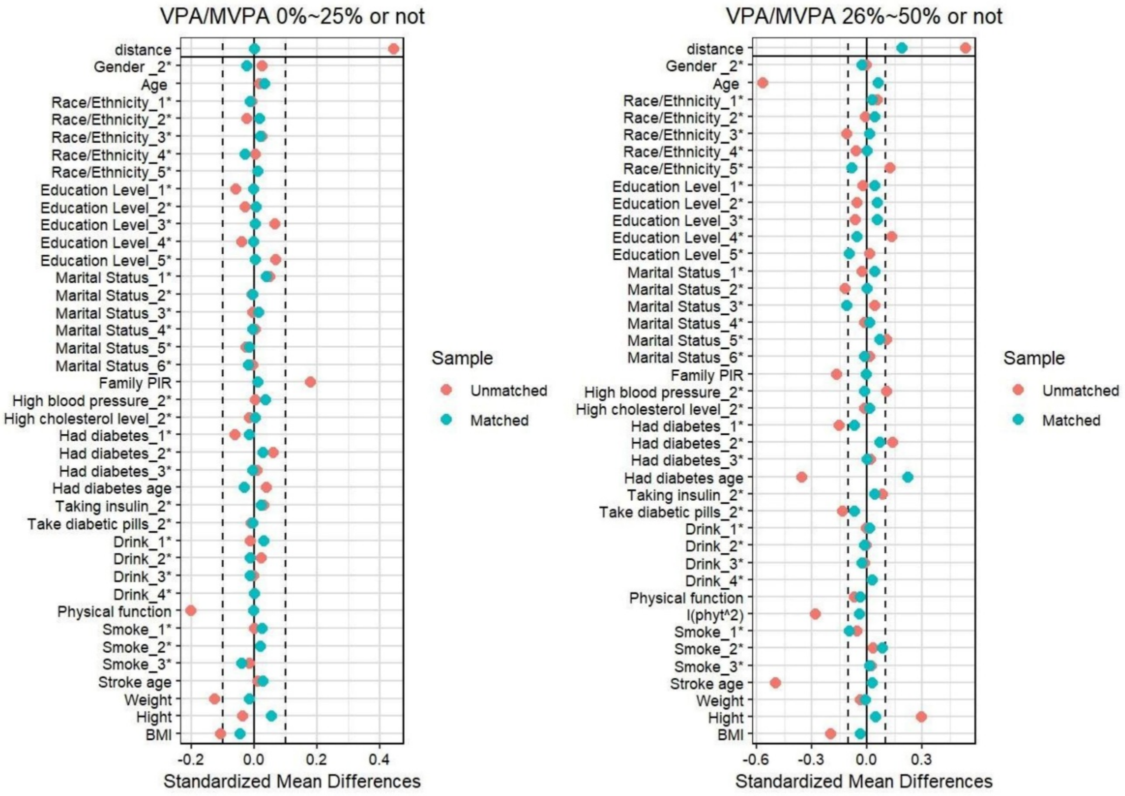

Fig. S7: The balance before and after matching7

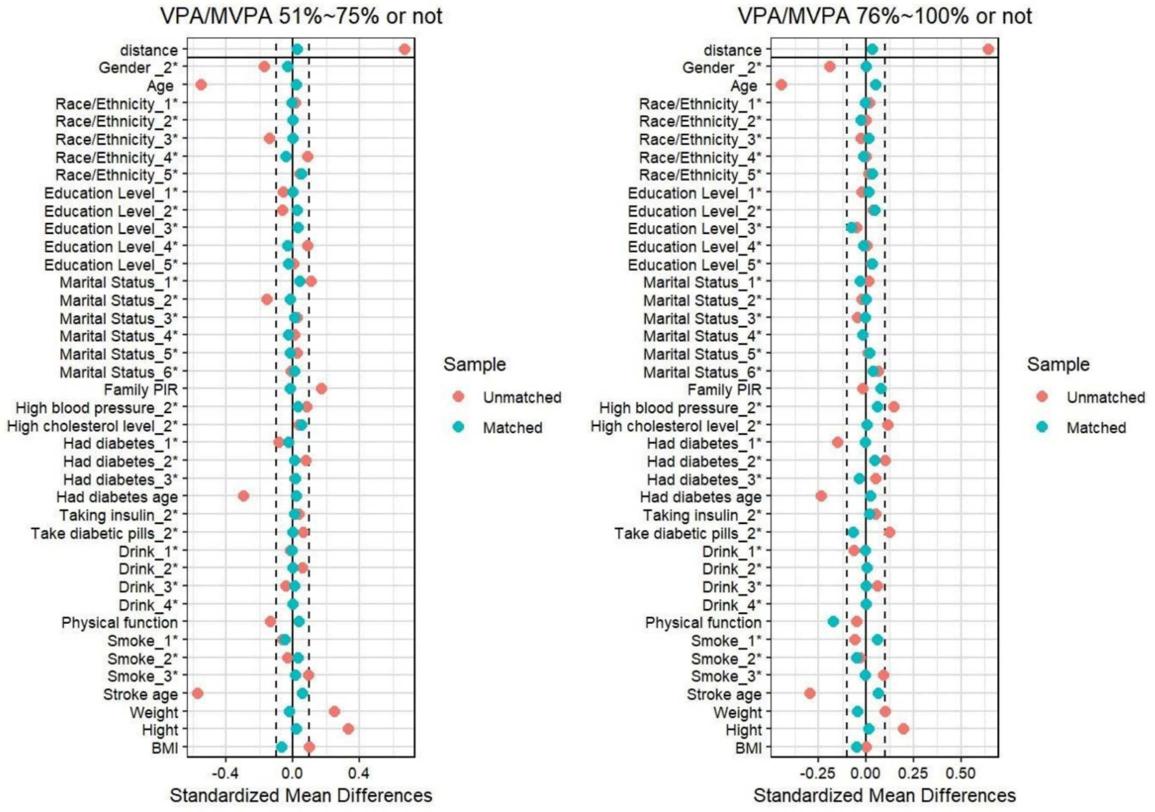

Fig. S8: The balance before and after matching8

*In additional figures 1-8, The specific implementation process of PSM is as follows. Logistic regression was used to calculate the PS score, and matching used 1:2 ratio sampling with replacement to select participants who did not perform relevant sports behaviors for matching, and nearest matching was used to obtain the final matching results. Each matching is carried out independently. Missing data was interpolated using k-Nearest Neighbor (K-NN) to ensure the progress of PSM. When analyzing whether the VPA duration is between 75 and 150 minutes, the nearest matching method has poor balance before and after matching. Consequently, full sample matching is selected. Poorly matched for stroke age (SMD = 0.1598) and PIR (SMD = 0.1247) only, but the V.Ratio of both is less than 2, the matching balance is acceptable.

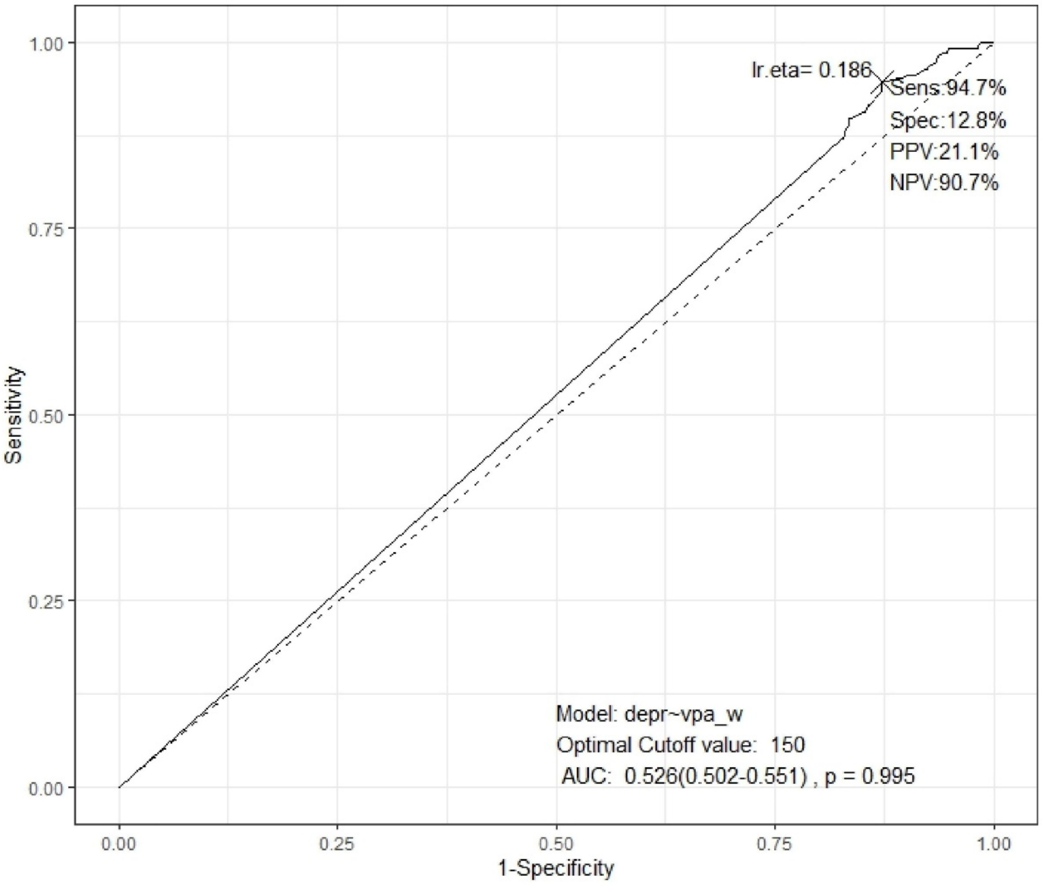

Fig. S9: The ROC plot of weekly VPA time

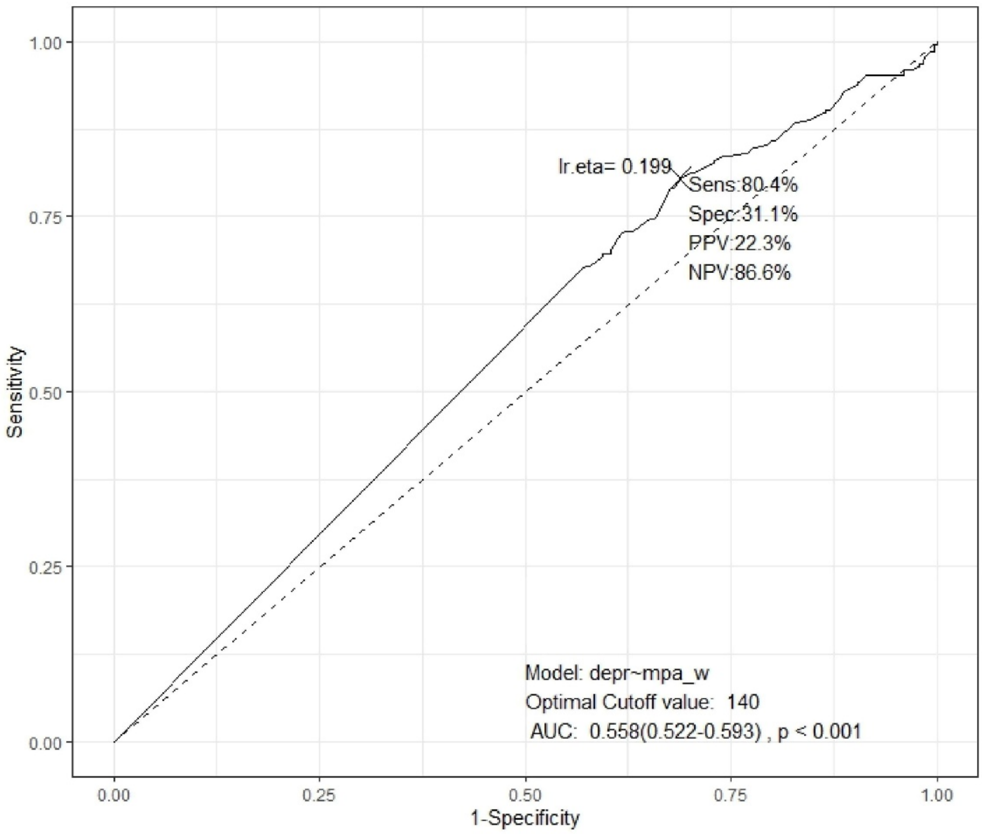

Fig. S10: The ROC plot of weekly MPA time

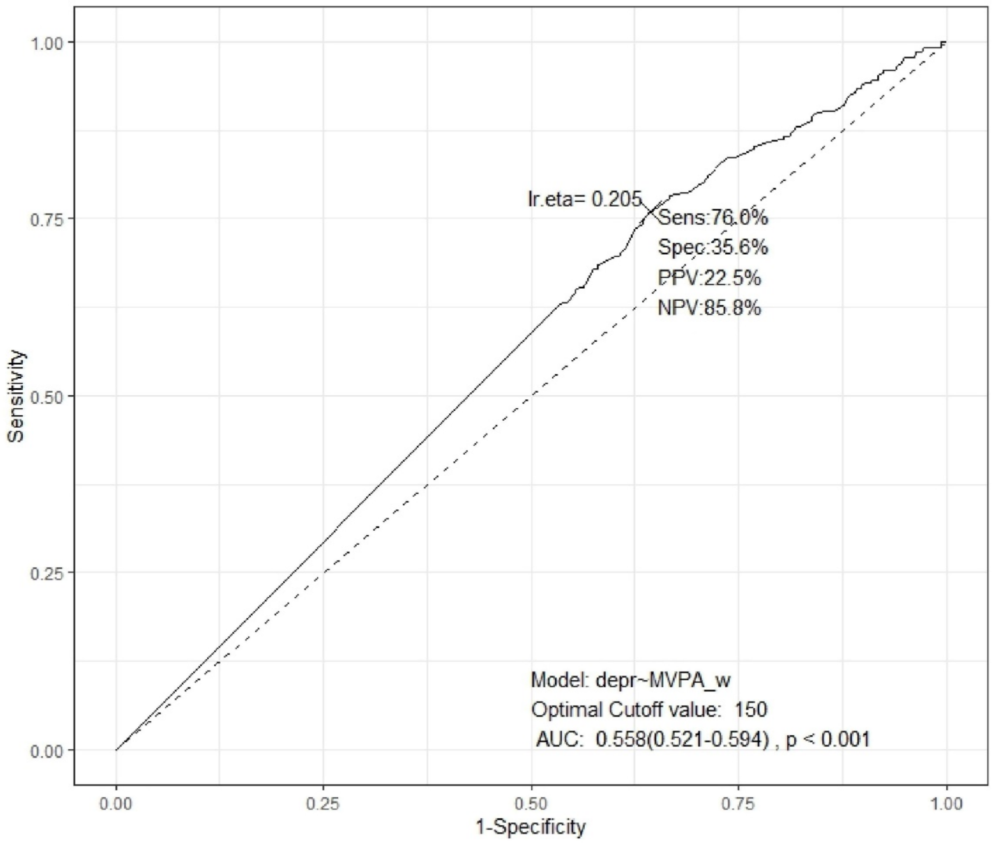

Fig. S11: The ROC plot of weekly MVPA time
